# The Effect of COVID-19 and Related Lockdown Phases on Young Peoples' Worries and Emotions: Novel Data From India

**DOI:** 10.3389/fpubh.2021.645183

**Published:** 2021-05-20

**Authors:** Meenakshi Shukla, Rakesh Pandey, Tushar Singh, Laura Riddleston, Taryn Hutchinson, Veena Kumari, Jennifer Y. F. Lau

**Affiliations:** ^1^Department of Psychology, Magadh University, Bodh Gaya, India; ^2^Department of Psychology, Banaras Hindu University, Varanasi, India; ^3^Department of Psychology, Institute of Psychiatry, Psychology and Neuroscience, King's College London, London, United Kingdom; ^4^Division of Psychology, Department of Life Sciences, and Center for Cognitive Neuroscience, College of Health, Medicine and Life Sciences, Brunel University, London, United Kingdom

**Keywords:** COVID-19, young people, India, worries, emotions

## Abstract

The COVID-19 pandemic has posed unprecedented stress to young people. Despite recent speculative suggestions of poorer mental health in young people in India since the start of the pandemic, there have been no systematic efforts to measure these. Here we report on the content of worries of Indian adolescents and identify groups of young people who may be particularly vulnerable to negative emotions along with reporting on the impact of coronavirus on their lives. Three-hundred-and-ten young people from North India (51% male, 12–18 years) reported on their personal experiences of being infected by the coronavirus, the impact of the pandemic and its' restrictions across life domains, their top worries, social restrictions, and levels of negative affect and anhedonia. Findings showed that most participants had no personal experience (97.41%) or knew anyone (82.58%) with COVID-19, yet endorsed moderate-to-severe impact of COVID-19 on their academics, social life, and work. These impacts in turn associated with negative affect. Participants' top worries focused on academic attainments, social and recreational activities, and physical health. More females than males worried about academic attainment and physical health while more males worried about social and recreational activities. Thus, Indian adolescents report significant impact of the pandemic on various aspects of their life and are particularly worried about academic attainments, social and recreational activities and physical health. These findings call for a need to ensure provisions and access to digital education and medical care.

## Introduction

The COVID-19 pandemic has had far-reaching consequences on the physical and mental health of individuals as well as the health of economies across the globe. While young people may be less susceptible to severe forms of the illness, suffering milder symptoms, lower morbidity, and better prognosis compared to adults (1, 2) they have experienced an upsurge in stress (3, 4) precipitating loneliness, anxiety and depression in many (5–8). As emotional symptoms in adolescence can become associated with many serious mental health outcomes including suicide, long-term physical health consequences, and significant healthcare burden (9–11), the effect of COVID-19 on young people's mental health could be more damaging in the longer run than the infection itself (12). Measuring early signs of mental health challenges such as worries and negative emotions in young people is thus an urgent priority for researchers (13, 14) as well as policy-makers, including identifying those most vulnerable to mental health difficulties. While this information is crucial for both high- and low-income countries, countries with lower resources dedicated to mental health may benefit more from early forecasts of these needs.

India has one of the highest COVID-19 infection rates in the world with over 2.5 million confirmed cases and the death toll on the rise (15, 16). The first case of COVID-19 was identified on January 30, 2020 in Kerala (17) in a student who had returned from Wuhan, China (18). However, since March 2020, there has been an upsurge in the spread of the infection. In response, the Government imposed a nationwide lockdown to prevent community transmission of the infection. Despite some regional differences in the extent of lockdown restrictions, based on total COVID-19 cases in that region (18), everyone in India has experienced closure of educational and training institutions; hotels and restaurants; malls, cinemas, gyms, sports centers; and places of worship. A recent correspondence article by Patra and Patro (19) speculated that school closures in particular may have been especially damaging for young people and highlighted the urgent need to address mental health issues in Indian adolescents. Yet there have been no such systematic efforts to our knowledge. Here, we report new data from a small cohort of young people from India. We describe their experiences of the COVID-19 pandemic and the impact of COVID-19 pandemic on their daily life. We describe the content of the most common worries reported by young people alongside quantitative measures of current negative and (absence of) positive emotions—symptom-markers of common mental health difficulties such as anxiety and depression. We then assess which young people (in terms of gender, age, and socioeconomic status) are particularly susceptible to reporting more negative emotions and fewer positive emotions. In India, before the pandemic started, public awareness around mental health in young people had been increasing along with the recognition that such problems can be economically costly (20). Our data can thus signpost emerging, potentially costly mental health problems post-pandemic.

## Method

### Participants and General Procedures

This study received approval from the Institutional Ethics Committee, Institute of Medical Sciences, Banaras Hindu University (Ref No.: Dean/2020/EC/1975) and King's College London Research Ethics Committee (Ref: HR-19/20-18250). Participants were recruited between June 5, 2020 and July 12, 2020. Prospective participants from different states of North India (Uttar Pradesh, Bihar, New Delhi, West Bengal, Madhya Pradesh, Gujrat) and their parents were identified by circulating information about the study including eligibility criteria (aged 12–18 years; currently residing in India) through social media sites, such as Facebook and WhatsApp. Interested and eligible individuals were sent bilingual (Hindi and English) information sheets (one for young people, one for the parents if the participant was aged 12–17 years). Those who agreed to participate after reading the information sheet received the survey link for both the English and Hindi versions and were requested to complete one based on their language preference. The survey link began with a question about the participants' age. If the participant was 18 years, they viewed and completed a consent form with an electronic signature and their contact details for follow-up assessments. Any participant aged 12–17 years was presented with an assent form with a parental/guardian consent form. To verify that parent/guardian consents were authentic, follow-up phone contact was made with the parent/guardian using the provided contact details. Survey questions were not presented further for incomplete consent/assent forms.

The online survey was developed using Qualtrics software (Qualtrics, Provo, UT). The first third of the survey comprised questions around demographics, personal experiences and knowledge of others who had been infected by the coronavirus, extent of social restrictions and social contact, and the impact of the viral outbreak on various life domains. The second third of the survey included measures of poor mental health such as negative affect, anhedonia (absence of positive affect), and the content of worries. The final third included measures of well-being (positive aspects of mental health), more specific negative emotional experiences (loneliness, boredom) and a cognitive measure (positive and negative future imagery) (presented elsewhere). All Hindi translations used the translation-back-translation method. MS completed the first set of translations, which were back translated by TS. JL checked the back-translations. Where there were definitional discrepancies with the original scale, these were discussed with RP and VK and re-translations were done by MS. The average time taken by the participant to complete the survey was 20 min.

### Measures

#### Demographics

Participants submitted information on their age, sex assigned at birth, family monthly income level, and number of family members.

### Personal Experiences of and Knowledge of Close Others With COVID-19

Five items (with yes/no responses) measured the extent to which participants had experienced the infection: have you ever been affected or suspected of having the coronavirus infection at any time, do you currently have a confirmed diagnosis of coronavirus infection, are you currently suspected of having a diagnosis of coronavirus infection, have you had a past confirmed diagnosis of coronavirus infection but have now recovered, have you had a past suspected diagnosis of coronavirus infection but have now recovered. Five items (with yes/no responses) assessed whether participants knew others who had experienced the infection, including: a family member, friend, other acquaintance (e.g., classmate), other individual known indirectly (e.g., acquaintance of a family member/friend/acquaintance), know no one with the illness. If the participants endorsed one of the first 4 items, they were asked whether the individual affected had recovered, were still recovering, were hospitalized or had passed away.

### Social Restrictions Associated With COVID-19

To describe the extent of reduced social contact, participants indicated the total number of days spent in self-isolation (i.e., not leaving the house), days in which they spent 15 min or more outside the house, days in which they had face-to-face contact with another person for 15 min or more, days in which they had a phone or video call with another person for 15 min or more.

### Impact of COVID-19

Participants rated the impact of the outbreak (including associated lockdown measures) on work, study, finances, social life (including leisure activities), relationship with family, physical health, emotions, and caring responsibilities (for children/siblings or elderly/fragile family members) over the last 2 weeks on a 5-point scale (0 = not applicable/none, 1 = very mildly, 2 = mildly, 3 = moderately, 4 = severely). Responses were summed across items to create a total impact score. In the current sample, the internal consistency reliability for the impact items was 0.706.

### Content of Worries

Participants were asked to write down their top 3 worries using free text boxes. All free text responses were reviewed by two researchers (MS, TS), who then independently derived “worry categories” based on these responses. The categories proposed by MS and TS were then reviewed by RP, VK, and JL. Where common categories were identified by both researchers these were used in the final worry categories. Where there were differences, these were resolved through discussions, using the life domains listed in the COVID-19 impact questions to help guide the identification of conceptually distinct areas. The final 12 categories along with their descriptions are shown in **Table 4**. Using this coding scheme and definitions, all responses were coded by both MS and TS independently to assess inter-rater agreement (Cohen's Kappa reliability). This was 0.98 for Worry 1, 0.90 for Worry 2, and 0.91 for Worry 3.

### Negative Affect

The 10 negative affect items from the Positive and Negative Affect Schedule (21) were used to assess negative emotions. Respondents used a 5- point Likert scale ranging from 1 (very slightly or not at all) to 5 (extremely) to indicate the extent to which they experienced the given mood states during the last 2 weeks. A total negative affect score, ranging from 10 to 50, was created by summing across the scores of individual items. Cronbach's alpha was 0.878.

### Anhedonia

Nine items (nos. 1, 3, 4, 5, 7, 9, 10, 13, and 14) from the 14-item Snaith-Hamilton Pleasure Scale (22) were used to index anhedonia, the inability to experience pleasure; the remaining 7 items were deemed unlikely to apply during lockdown phases. Four response options were given for each item (strongly disagree, disagree, agree, or strongly agree), where strongly disagree and disagree were scored 1 and agree and strongly agree, scored 0. A summed score across items therefore ranged from 0 to 14, where higher scores indicated greater absence of positive affect. Cronbach's alpha was 0.723.

### Statistical Analyses

After presenting the demographic characteristics of the sample, gender differences in age and income were analyzed using independent sample *t*-tests. Descriptives of young peoples' personal experiences of the infection, knowledge of others with the infection, the effect of lockdown on social isolation and contact with others and impact across other life domains were presented next. Before conducting any statistical analysis, the data were checked for fulfilling the assumptions for normality (23). The data did not show serious deviations from normality based on the histogram plots, except a slight positive skew for anhedonia. The skewness and kurtosis values of the data were also within the recommended limit of ±2 (24, 25), most being < 1 (except for anhedonia which was >1). Thus, we employed parametric analyses for all the variables except for anhedonia which was explored using non-parametric tests. We investigated the degree to which the overall impact of COVID-19 across life domains varied as a function of gender (using independent samples *t*-test) and age and family income levels (using bivariate correlations). For the worry data, the percentage of individuals endorsing each worry category was calculated for each of the top 3 worries (first, second, third). However, in the final analysis, we collapsed across the top 3 worries to generate an overall percentage across participants of endorsing that worry among one of their top 3 worries. This meant, for instance, that any participant who rated the same worry across all 3 of their top worries was only represented once. The final percentage of young people endorsing the worry categories was compared across gender and for interpretability, by categorical age groups (Younger adolescents = 12–15 years; Older adolescents = 16–18 years) using chi-square tests. Finally, we presented data on negative affect and absence of positive affect (anhedonia); we investigated how these variables varied across gender, age, and per capita monthly income using multiple linear regression models; we further assessed whether inclusion of interaction terms significantly added to variance explained. Given a slight positive skew for anhedonia, we log-transformed this variable when conducting the regression analysis. To complement the multiple regression analysis of demographic predictors and their interactions, we also ran a series of parametric and non-parametric *t*-tests and correlations for negative affect and anhedonia, respectively, to assess the extent to which gender, age and family income levels individually associated with these variables. Correlations also assessed the extent to which the overall impact of COVID-19 associated with negative affect and anhedonia.

## Results

### Demographic Characteristics

The final sample comprised 310 Asian-Indian adolescents (Mean age = 15.69 years; SD = 1.92) of whom 159 were males (Mean age = 15.60 years; SD = 1.98) and 151 were females (Mean age = 15.78 years; SD = 1.87). Males and females did not differ significantly in age, *t*_(308)_ = −0.84, *p* = 0.40, *d* = 0.05. Furthermore, the Levene's test of equality of variances indicated an equal spread of scores in males and females (*F* = 0.89, *p* = 0.34). Only 192 participants provided data for monthly per capita family income, which ranged from 125 to 150,000 Rupees (Mean = 9698.20; SD = 18315.22) with no significant mean or variance differences in the monthly per capita income between males and females [Male Mean = 8343.61; SD = 15065.95; Female Mean = 11439.82; SD = 21768.30; *t*_(190)_ = −1.16, *p* = 0.25], *d* = 0.16, Levene's test of equality of variances: *F* = 2.63, *p* = 0.10.

### Experiences of COVID-19

Item-level data for personal experiences and knowledge of close others with COVID-19 infections are presented in Table 1 for all participants; and males and females separately. Most young people had not personally experienced or known someone with the coronavirus infection. Of those who did report knowing someone infected with COVID-19, just under half (49.09%) reported that the affected person they knew had recovered from the infection, 12.73% reported that the person was still recovering, 14.54% reported that the known person was hospitalized, while 25.45% participants reported that the affected person passed away.

**Table 1:** Personal experience of and knowledge of others with COVID-19 (Of note, while the first set of questions about personal experiences of COVID-19 reflects mutually exclusive response options (therefore adding up to 100%), the set of questions around knowledge of others are not all mutually exclusive. For instance, a participant reporting a family member as well as an acquaintance infected with the virus would be included twice, once when calculating the percentage of participants reporting an infected family member and once when calculating the percentage of participants having an infected acquaintance. Therefore, participants having knowledge of others with COVID-19 do not add up to 100%).

**Personal experience of COVID 19 (% of sample)**	**Overall sample (*N* = 309)**	**Males (*N* = 158)**	**Females (*N* = 151)**
Neither affected nor suspected of coronavirus infection at any time	97.41	96.20	98.68
Current confirmed diagnosis of coronavirus infection	0.32	0.63	0
Current suspected diagnosis of coronavirus infection	0.32	0.63	0
Past confirmed diagnosis and now recovered	0.65	1.26	0
Past suspected diagnosis and now recovered	1.30	1.26	1.32
**Knowledge of others with COVID 19 (% of sample)**	**Overall sample (*****N*** **= 310)**	**Males (*****N*** **= 159)**	**Females (*****N*** **= 151)**
No knowledge of others	82.58	82.39	82.78
Family member	2.63	3.14	1.26
Friend	0.64	0.63	0.66
Other acquaintance	4.52	5.03	3.97
Any other person	11.29	10.69	11.92

### Social Restrictions and Impact of COVID-19

Item-level data for questions around social restrictions and reduced social contact are presented in Table 2 for all participants, for male and females separately; and correlations with age and monthly per capita family income. Compared to males, female participants spent significantly more days in self-isolation and more days engaging in phone or video call for 15 min or more. Participants with lower monthly per capita income spent more days in which they were out for 15 min or more, but fewer days engaging in phone or video calls. Age did not correlate with perceived social restrictions.

**Table 2:** Restrictions associated with COVID−19.

**Restrictions associated with COVID 19 (in the last 2 weeks)**	**Overall mean (SD)**	**Levene's statistic (*F*, *p*-value)**	**Males mean (SD)**	**Females mean (SD)**	***t* (*p*-value), Cohen's d df = 308**	**Age *r* (*p*-value)**	**Monthly per capita family income *r* (*p*-value)**
Days spent in self−isolation (not leaving the house)	9.01 (4.85)	0.11 (0.74)	**8.28 (4.82)**	**9.77 (4.78)**	**−2.75 (0.01), 0.31**	0.020 (0.68)	0.04 (0.58)
Days on which spent 15 min or more outside the house	5.52 (4.35)	0.14 (0.70)	5.95 (4.23)	5.07 (4.43)	1.80 (0.07), 0.21	0.048 (0.40)	**−0.16 (0.03)**
Days on which had face−to−face contact with another person for 15 min or more	5.87 (4.73)	6.02 (0.02)	157.89 (159)^#^	152.99 (151)^#^	11625.00 (0.63)^*^	0.006 (0.91)	−0.03 (0.71)
Days on which had phone/video call with another person for 15 min or more	8.23 (4.60)	0.11 (0.73)	**7.68 (4.60)**	**8.80 (4.56)**	**−2.15 (0.03), 0.24**	−0.008 (0.89)	**0.15 (0.04)**

# *Mean Rank (N). Values in bold indicate significant means and statistical test results*.

* *Mann-Whitney U (p-value)*.

Mean ratings of the impact of COVID-19 on various life domains are presented in Table 3. Looking at how many young people endorsed moderate-to-severe impact for each domain, 43.6% reported this on their work, 56.8% on their studies, and 48.4% on their social life and recreational activities. Just under half of young people reported moderate-to-severe impact of the pandemic on their family relationships (48.4%), on their caring responsibilities (49.4%) and on their physical health (42.6%). However, 52% reported this for their emotions. For finances, moderate-to-severe impact was reported by 26.8% of young people. Sex, age, and per capita monthly income effects were examined on each domain-specific impact score and the total score, summed across mean ratings for each domain (Table 3). No significant associations emerged between age and impact across any domain (Table 3). Males reported higher mean impact scores for relationships with family members and physical health. Participants with lower per capita income experienced more impact of COVID-19 across life domains (indicated by total impact score) than those with higher monthly per capita income.

**Table 3:** Impact of COVID-19 on psychosocial domains.

**Impact of COVID 19 on psychosocial domains (5-point scale; 0 = not applicable/none, 1 = very mildly, 2 = mildly, 3 = moderately, 4 = severely)**	**Overall mean (SD)**	**Levene's statistic (F, *p*-value)**	**Males mean (SD)**	**Females mean (SD)/mean rank**	**t (*p*-value), Cohen's d df = 308**	**Age r (*p*-value)**	**Monthly per capita family income r (*p*-value)**
Work	1.91 (1.56)	2.56 (0.11)	2.00 (1.61)	1.81 (1.50)	1.08 (0.28), 0.12	0.018 (0.75)	−0.05 (0.47)
Study	2.45 (1.41)	4.07 (0.04)	150.66 (159)^#^	160.60 (151)^#^	11235.00 (0.32)^*^	0.029 (0.61)	0.01 (0.89)
Finances	1.33 (1.46)	0.12 (0.73)	1.35 (1.46)	1.30 (1.46)	0.33 (0.74), 0.03	−0.003 (0.96)	−0.01 (0.87)
Social life (including family activities)	2.13 (1.38)	1.92 (0.17)	2.06 (1.41)	2.20 (1.35)	−0.91 (0.37), 0.10	0.084 (0.14)	0.08 (0.26)
Relationship with family	2.04 (1.64)	0.01 (0.91)	**2.28 (1.63)**	**1.78 (1.61)**	**2.73 (0.01), 0.31**	−0.102 (0.07)	**−0.26 (0.00)**
Physical health	1.91 (1.47)	1.79 (0.18)	**2.11 (1.51)**	**1.70 (1.40)**	**2.48 (0.01), 0.28**	0.028 (0.62)	**−0.16 (0.03)**
Emotions	2.19 (1.45)	0.01 (0.93)	2.30 (1.45)	2.07 (1.45)	1.35 (0.18), 0.16	−0.051 (0.37)	−0.12 (0.10)
Caring responsibilities for children/siblings, elderly/other adults who may have long−term health problems	2.07 (1.53)	0.02 (0.89)	2.08 (1.53)	2.06 (1.54)	0.09 (0.93), 0.01	−0.003 (0.96)	−0.12 (0.10)
Summed score of psychosocial impact of COVID	16.01 (6.81)	5.80 (0.02)	162.53 (159)^#^	148.09 (151)^#^	10886.00 (0.16)^*^	−0.00 (0.96)	**−0.14 (0.05)**

# *Mean Rank (N). Values in bold indicate significant means and statistical test results*.

* *Mann-Whitney U (p-value)*.

### Content of Worries

The percentages of young people endorsing each worry category for each of their top 3 worries are presented in the first three columns of Table 4. These were used to derive the overall percentages of young people endorsing each worry category *as one of their top 3 worries* presented in Column 4. Using this fourth column, we noted that most participants reported education and studies (Academic) as one of their top worries. The second most common worry of participants centered around “Physical health, fitness, and safety.” Worries about “Social and recreational activities” also emerged as a major concern for several participants, followed by “Finances.” Some participants also listed “Global and societal concerns.” More females reported concerns about “Academic,” and “Physical health, fitness, and safety,” compared to males (Table 4) while male participants reported more worries around “Social and recreational activities” activities than female participants. Comparison of worries across the adolescent groups revealed that while a higher percentage of older adolescents reported each of the worries as one of their top three worries compared to younger adolescents (except for “Unclear” category), the differences were statistically significant only for “Academic,” “Physical health, fitness, and safety,” “Global and societal concerns,” and “Other” categories (Table 4).

**Table 4:** Participants' reported content of top three worries over the last 2 weeks.

**Worry categories**	**Description of worry categories**	**Percentage of participants reporting the worry**			**Percentage of participants reporting the worry as one of their top worries**						
		**Top worry 1**	**Top worry 2**	**Top worry 3**							
		**Overall**	**Overall**	**Overall**	**Overall**	**Males**	**Females**	**χ^**2**^**	**Younger**	**Older**	**χ^**2**^**
		**(*N* = 307)**	**(*N* = 307)**	**(*N* = 307)**				**(*p*-value)**	**adolescents**	**adolescents**	**(*p*-value)**
Academic	Concerns around education: status of current studies, examinations, college admissions, online classes, studying at home	47.2	30.9	15.6	**73.5**	66.7	80.8	**7.82 (0.01)**	40.35	59.65	**8.15 (0.01)**
Future career	Longer-term concerns around job opportunities, training and employment	4.9	3.3	3.3	11.3	10.1	12.6	0.48 (0.49)	37.14	62.86	2.11 (0.15)
Finances	Concerns around short-term money problems faced/anticipated by participants about themselves and their family: paying the rent, family businesses, salary reductions	4.6	8.5	11.7	**23.5**	24.5	22.5	0.17 (0.68)	42.46	57.53	1.60 (0.21)
Food and available resources	Concerns about immediate/long-term food supplies and other essential resources	1.0	2.3	1.3	4.2	5.0	3.3	0.55 (0.46)	46.15	53.85	0.07 (0.79)
Physical health, fitness and safety	Concerns about being infected during the pandemic, health of family and friends, more general concerns about physical fitness	14.3	14.7	20.5	**40.6**	35.2	46.4	**3.98 (0.04)**	36.51	63.49	**8.48 (0.01)**
Family relationships	Concerns around managing conflicts with/between other family members	1.0	2.6	2.0	5.5	4.4	6.6	0.72 (0.40)	41.18	58.82	0.48 (0.49)
Social and recreational activities	Concerns around the lack of socializing/social activities, typical sporting and leisure activities/entertainment	7.2	12.1	18.6	**30.3**	37.1	23.2	**6.99 (0.01)**	47.87	52.13	0.17 (0.68)
Mental health and emotions	Concerns about psychological symptoms/intense emotional experiences: nightmares, sleeplessness, boredom, guilt, loneliness	0.0	2.6	3.6	5.8	6.9	4.6	0.74 (0.39)	33.33	66.67	1.70 (0.19)
Global and societal concerns	Concerns about country's economy, migrant labor problems, poor people, death rates and bereavements globally, future of the world	6.2	12.4	9.4	**23.2**	19.5	27.2	2.54 (0.11)	36.11	63.89	**5.09 (0.02)**
Unspecified COVID and lockdown related uncertainties	Concerns indicating uncertainty owing to the course of the pandemic and future lockdown phases	6.8	2.0	1.6	9.0	8.2	9.9	0.27 (0.60)	35.71	64.28	2.04 (0.15)
Other	Concerns where the content of the worry was ambiguous/did not fall into a specific category, e.g., buying a new phone, getting a new cycle, marriage	1.6	4.2	5.9	10.0	12.6	7.3	2.38 (0.12)	22.58	77.42	**6.94 (0.01)**
Unclear	Responses such as “don't know” or “no problems”	5.2	4.6	6.5	9.0	10.1	7.9	0.45 (0.50)	60.71	39.28	1.19 (0.28)

*Values in bold indicate significant means and statistical test results. The five highest overall percentage of individuals reporting a particular worry are also put in bold*.

### Negative Affect

A stepwise multiple regression was conducted with negative affect as the dependent variable and age, gender, and per capita monthly income as predictors in step 1 and their interaction terms (i.e., age x gender, age x per capita monthly income, gender x per capita monthly income, and age x gender x per capita monthly income) entered in step 2. Results indicated that the model predicted by the demographic variables was non-significant, *F*_(3,187)_ = 2.11, *p* = 0.10 (Adjusted *R*^2^ = 0.017). Nor did the inclusion of interaction terms significantly increase variance explained, *R*^2^ change = 0.004, *p* = 0.36, *F*_(4,186)_ = 1.79, *p* = 0.13 (Adjusted *R*^2^ = 0.016). These findings suggested that males and females did not differ on total negative affect, *t*_(305)_ = −0.90, *p* = 0.37, *d* = 0.10 [Male mean = 21.67 (SD = 8.78), Female mean = 22.51 (SD = 7.85)], Levene's test of equality of variances: *F* = 0.46, *p* = 0.50. Nor were there significant correlations with age (*r* = 0.09, *p* = 0.10) and per capita monthly income (*r* = −0.11, *p* = 0.13). However, significant correlations emerged between negative affect and reported impact of COVID-19 across life domains (*r* = 0.26, *p* < 0.001). Negative affect correlated (mostly) weakly but significantly with impact of COVID-19 on social life (*r* = 0.13, *p* = 0.02), relationship with family (*r* = 0.14, *p* = 0.01), physical health (*r* = 0.20, *p* < 0.001), emotions (*r* = 0.23, *p* < 0.001), and caring responsibilities (*r* = 0.18, *p* < 0.001), but not with work (*r* = 0.11, *p* = 0.06), study (*r* = 0.07, *p* = 0.22), and finances (*r* = 0.11, *p* = 0.06).

### Anhedonia

A stepwise multiple regression, similar to that conducted for “negative affect” was conducted for anhedonia but with the log-transformed scores since the anhedonia scores were slightly positively skewed. Results showed that the model with all demographic predictors was non-significant, Model 1: *F*_(3,156)_ = 1.44, *p* = 0.23 (Adjusted *R*^2^ = 0.008). Inclusion of interaction terms did not significantly increase the variance explained, *R*^2^ change = 0.000, *p* = 0.85, *F*_(4,155)_ = 1.08, *p* = 0.37 (Adjusted *R*^2^ = 0.002). Assessment of the individual demographic predictors showed that males (Mean Rank = 165.43) reported higher levels of anhedonia than females (Mean Rank = 141.09); Mann–Whitney *U* = 9838.50, N1 = 156, N2 = 150, *p* = 0.01. Participants belonging to families with higher monthly per capita income experienced lower levels of anhedonia (*r*_s_ = −0.17, *p* = 0.02). However, there were no significant correlations between reported impact summed across life domains and anhedonia (*r*_s_ = −0.02, *p* = 0.74). While anhedonia correlated positively but weakly with impact of COVID-19 on physical health (*r*_s_ = 0.13, *p* = 0.02), it showed a significant but weak negative relationship with impact of COVID-19 on study (*r*_s_ = −0.20, *p* < 0.001) and social life (*r*_s_ = −0.11, *p* < 0.05). Anhedonia did not correlate significantly with the impact of COVID-19 on work (*r*_s_ = 0.01, *p* = 0.93), finances (*r*_s_ = −0.02, *p* = 0.70), relationship with family (*r*_s_ = 0.09, *p* = 0.13), emotions (*r*_s_ = −0.04, *p* = 0.45), and caring responsibilities (*r*_s_ = −0.02, *p* = 0.73).

## Discussion

This paper describes baseline data for a cohort of Indian adolescents recruited to a study aiming to assess the longitudinal impact of COVID-19 on negative emotions, worries and strategies used to manage these emotions. Participants were recruited at a time when the total number of coronavirus-infected people in India stood at 236,184 and ended when the total number of infections was 879,466, showing a consistent rise during the period of (baseline) data collection (16). Yet, even during this period of rising infections, personal experiences and knowledge of others who had been exposed to the coronavirus infection were uncommon for most of our participants. Nonetheless, participants reported moderate-to-severe impact of COVID-19. The impact data together with qualitative data on their top worries, underscored academic studies as a salient area of concern for most young people in this cohort, a likely outcome of social distancing measures preventing school attendance and educational progress. Other salient worries for young people were concerns over the health and safety of self and loved ones and the absence of age-typical social and recreational activities, again expected worries emerging due to the pandemic itself and associated lockdown measures. Interestingly, young people commonly reported worries for their own finances as well as the Indian and global economy, and society more generally. Significantly higher percentage of older adolescents (16–18 years) than younger ones (12–15 years) were worried about their academics, physical health and safety, global and societal concerns and other kinds of worries, which can be expected since with increasing age, the academic work and curriculum gets more difficult and late adolescence is also the crucial time for career explorations (26). Adolescence is a time of emerging independence (taking on more responsibilities for their own future) but also of interdependence, where self-construal becomes linked to roles and commitments to other groups in society (27). Identifying the content of these stressors and worries can help governments decide where to propose subsequent policy changes and facilitate society-wide measures. Beyond the need for dedicated mental health services (helplines, centers) called for in earlier papers [e.g., (28)], our data specifically underscore the need for investment of resources into the safe opening of schools, changes to the curriculum and/or the provision of digital education to all young people. Reassurance over access to quality medical care is also a priority.

Within these impacts and worries, there were some gender differences. More females than males reported Academic as a top worry (though this gender difference was not replicated in quantitative impact ratings), which is likely since Indian adolescent females have been reported “more sincere” toward studies than Indian adolescent males, potentially meaning they are more committed and motivated to academic achievement (29). Males reported a greater impact of COVID-19 on physical health in quantitative ratings; in the Indian context male adolescents are more likely to engage in outdoor sports (30) and experience fewer sociocultural barriers to outdoor physical activity (31) than female adolescents. This difference between genders where males spent more time out of the house than females, may also have emerged because males identified social and recreational activities as a top concern; females by contrast, followed restrictions associated with COVID reporting more days in social isolation and on phone/video calls. Perhaps relatedly, more females expressed worries over physical health, fitness, and safety from contracting the virus than male participants. Sedentary lifestyles resulting from the lockdown (32) may not only affect childhood obesity but can also significantly affect mental health of adolescents. Some interesting trends were also noted in relation to socio-economic status (SES) of the participants, as indexed by the per capita monthly income of their families. Lower SES was associated with a higher impact of COVID across life domains but particularly with impacts on physical health and family. Lower SES was associated with more days participants spent outside of the home, which could explain the reported impact on physical health. Adolescents belonging to lower SES may be residing in crowded living situations, which together with parental stress due to the economic crisis (33), may mean them having to navigate more complicated family dynamics. Higher SES was associated with more days spent on phone/video calls, probably because participants belonging to higher SES have greater access to laptops, smartphones, and/or tablets than those from lower SES.

In terms of negative and (absence of) positive emotions, means reported in our sample using translated versions of standardized questionnaires were commensurate with those reported in general youth population samples in the west (34). Self-reported negative affect did not correlate with age, SES and did not vary between males and females but was greater in those reporting more impact of COVID-19 across life domains. Males and those from lower SES reported more anhedonia. These findings pursued longitudinally in time can help us to identify those who show propensity for anxiety/depression across time allowing us to signpost need for mental health resources. Although anhedonia was negatively linked with the impact of COVID-19 on study and social life of the participants, these associations were weak.

There are several study limitations. First, the sample has been obtained using convenience sampling methods (using social media) and responders were only from a few North Indian states. Hence it is difficult to say how representative it is of 12–18 year old Indian adolescents. Moreover, given the study survey requirements, only participants who had access to the Internet and had a registered phone number (to verify parental consent) could be recruited, biasing the study sample composition. However, SES classes seemed to be adequately represented since using the Modified BG Prasad Socio-economic Classification 2019 (35), (although there was some missing data) the sample reflected the entire continuum of SES classes in India. Second, as data was collected online, qualitative responses were unprobed and very often single word answers had to be coded, affecting the reliability of these data. Nonetheless, inter-rater reliability using this coding scheme was high. Third, participants did not report on whether they lived in rural or urban areas of their respective cities, and therefore our data cannot speak to rural-urban differences in adolescents' worries, negative and positive emotions. Future studies should measure and compare the impact of rural and urban populations on these indices of poor mental health. Finally, many of the scales used were not standardized. However, as internal consistencies were acceptable, this study adds potential new measures for future studies of young people in the Indian context.

## Conclusions

Our study showed that even though a handful of participants had personal experiences of or knew someone who had been infected by COVID-19, all our participants reported considerable impact of the pandemic on various aspects of their life, which was linked to higher negative affectivity. Adolescents also expressed worries about their studies, physical health and safety as well as social and recreational activities, with some gender differences. While our findings are unable to demonstrate causality between the impact of these COVID-19 related changes and worries, negative affect and anhedonia, nonetheless, the findings highlight the urgent need for government policy makers to take concrete steps to mitigate potential adverse effects of the pandemic on the mental health of Indian adolescents.

## Data Availability Statement

The raw data supporting the conclusions of this article will be made available by the authors, without undue reservation.

## Ethics Statement

The studies involving human participants were reviewed and approved by Institutional Ethics Committee, Institute of Medical Sciences, Banaras Hindu University (Ref No.: Dean/2020/EC/1975); King's College London Research Ethics Committee (Ref: HR-19/20-18250). Written informed consent to participate in this study was provided by the participants' legal guardian/next of kin.

## Author Contributions

JL, MS, VK, RP, TH, LR, and TS contributed to the conception and design of the study. RP, TS, JL, VK, and MS contributed to the development of study materials, contributed to analysis, and interpretation of study data. MS and TS contributed to acquisition of study data. MS and JL wrote first draft of the paper. VK, RP, TS, TH, and LR critiqued the output for important intellectual content. All authors contributed to the article and approved the submitted version.

## Conflict of Interest

The authors declare that the research was conducted in the absence of any commercial or financial relationships that could be construed as a potential conflict of interest.
